# A new method for biological synthesis of agriculturally relevant nanohydroxyapatite with elucidated effects on soil bacteria

**DOI:** 10.1038/s41598-019-51514-0

**Published:** 2019-10-21

**Authors:** Ayushi Priyam, Ratul Kumar Das, Aaron Schultz, Pushplata Prasad Singh

**Affiliations:** 1 National Centre of Excellence for Advanced Research in Agricultural Nanotechnology, TERI - Deakin Nanobiotechnology Centre, Sustainable Agriculture Division, The Energy and Resources Institute (TERI), DS Block, India Habitat Centre, Lodhi Road, New Delhi, 110003 India; 2 School of Life and Environmental Sciences, Deakin University, Geelong, Victoria 3217 Australia

**Keywords:** Environmental impact, Nanoparticles

## Abstract

The study describes a novel and environment friendly route of biosynthesis of nanohydroxyapatite (nHAP). *Bacillus licheniformis* mediated synthesis of nHAP has been carried out with different phosphate concentrations (2%, 5%, 10% and 20% *w/v*) of potassium dihydrogen orthophosphate monobasic (K_2_HPO_4_). The synthesis is supported by a two-step mechanism – (i) solubilization of P by organic acids extracellularly secreted by the bacterial strain and (ii) gelation of P and Ca. The nHAP particles were characterized using electron microscopy and XRD analysis. Powdered crystalline particles with a size range of 30 ± 5 nm were obtained with shape and size dependent on phosphate concentrations. The particles showed no adverse effect on plant growth-promoting bacteria. Evaluation of nHAP prepared by this route with 2% P source provides scope for a wide range of applications, especially as a nano-fertilizer.

## Introduction

Global phosphorous (P) reserves are getting depleted rapidly and according to available evaluations, there will be no soil P reserve left by the year 2050^1^. Due to the continuous depletion of P in soil, only 40% of world’s arable lands have the effective crop yield^1^. According to estimates, 49% and 45% of the agricultural soils in India fall in the low and medium phosphorus content categories, respectively^2^. Therefore, to achieve a high-yield in agriculture, it is necessary to apply P-fertilizer in appropriate quantity in agriculture fields to deal with nutrient deficiency. The challenge to meet the increasing P demand in a sustainable manner at a global scale requires development of smart solutions. These new solutions must address the need for efficient P utilization; remediation, modifications and improvisations in the chemical properties of fertilizers; P fertilizer regulation and governance. In order to overcome the issues of run off, an attempt has been made to introduce less soluble forms of P fertilizers, such as those derived from synthetic apatite forms, Ca_5_(PO_4_)_3_X, (X = F, Cl, Br, or OH)^3^. These are advantageous in terms of lower availability to algae thereby reducing the possibilities of eutrophication. Conversely, the major disadvantage of such solid forms of P fertilizer is reduction in plant-uptake^3^. With emerging trends in agricultural nanobiotechnology, a scientifically viable solution may be proposed. Naturally occurring rock-phosphate is used traditionally as a phosphorus fertilizer but has low solubility. Increase in P solubility may be achieved through its nanoparticle formulation^4^. Yet, the nanoparticle formulation from rock phosphate would be a complex of a number of contaminant elements that are present in the parental material, instead of a pure P fertilizer.

The other conventional approaches for production of P-fertilizer through use of non-renewable phosphate resources (phosphorite and rock phosphate), mineral acids (sulfuric acid) are expensive and generate many hazardous byproducts^5^. Possibility to obtain liquid bio-fertilizer by utilizing the waste from P rich raw materials such as bones and sewage sludge, via a solubilization process performed by microorganisms that are naturally present in the soil has also been demonstrated^6^. Labuda *et al*.^7^, confirmed that in the processes mediated by the microorganisms, the yield of solubilization from P by-products was higher (up to 80%) when compared with the utilization of phosphorite as a source of P (solubilization degree about 20–40%)^7^. However, synthesis of P-rich liquid bio-fertilizers using bones and sewage sludge can result in secondary environmental contaminants^8^.

Hydroxyapatite (HAP) has also been suggested as a good source of P-fertilizer. HAP is an essential bone mineral, and therefore, is a well-known biomaterial that has been explored for a variety of biomedical applications such as tissue engineering, dental implants and bone grafting^9,10^. Furthermore, nano formulation of HAP, nanohydroxyapatite (nHAP), too has been previously reported as a potent fertilizer at the lab scale^4,6,11^. nHAP has been reported to supply sufficient P nutrients to crop plants^4,11,12^. Unlike soluble phosphate salts and solid phosphates, nHAP shows lesser mobility to the surrounding agricultural and aqua-cultural areas, and therefore possesses a remarkable potential to enhance agronomical yield and reduce risks of water eutrophication^4,11,13^.

There are several methods for synthesizing nHAP as reported in the literature^14^, including wet chemical deposition, biomimetic routes, sol-gel, and hydrothermal. These conventional methods involve overt use of numerous chemicals to achieve controlled synthesis, but the synthesized nanomaterials often have high toxicity^14^. Therefore, current nanobiotechnology is focusing on environmentally benign biosynthesis processes^15^ in which microorganisms such as bacteria and fungi show potential as bio-manufacturing units to carry out nano-synthesis^15,16^. Biological systems have a unique ability to control the structure, phase, orientation and nano-structural topography of inorganic crystals, called bio-mineralization. Recent researches^17,18^ have shown the significance of biosynthesis of nanoparticles, particularly those of silver^19,20^, gold^21,22^ and titania^23^. Similarly, for preparing HAP nanostructured powders and coatings, bio-mineralization is categorized as one of the biomimetic routes that has many advantages in comparison with other solid state and wet chemical methods^24^. This method uses either cell free extracts or biomasses of biological organisms such as bacteria, fungi and microalgae, which are both economic and environmentally safe. It is also possible to control shape and size of nanoparticles in the biosynthetic processes by optimization of various factors such as pH, temperature, stirring rate and sintering temperature^25,26^. Although the bio-mineralization method has many advantages in comparison with several other possible routes of nHAP production, only a few reports on utilization of this method for synthesis are available in literature^24,27,28^.

In the present study, culture supernatants of non-pathogenic, phosphorus solubilizing bacterial species, *Bacillus licheniformis*, was used to synthesize nHAP. *B. licheniformis* like other *Bacillus* species has natural ability to release phosphorus from unavailable complexes^29,30^, primarily by releasing various organic acids^31–33^. Sharma *et al*. (2015), demonstrated the P solubilization potential of *B. licheniformis* when fed with phosphate-calcium minerals^32^. The two important factors that have been emphasized during the development and optimization of new synthesis methods include ease of mass production and safety in handling the bacterium. In the present study, the derived synthesis of nHAP was carried by using dipotassium hydrogen phosphate (K_2_HPO_4_) and calcium chloride (CaCl_2_) as precursor molecules and cell-culture of *B. licheniformis* as a nano-conversion tool. Complete physicochemical characterization of the synthesized nHAP was done. Our method demonstrated a completely novel route of nHAP fabrication by using certain P concentrations and studying the rate of P-solubilization by *B. licheniformis* while bio-mimicking the classical colloidal nanoparticle synthesis. Here, the nHAP particles are developed for their application as P-nanofertilizer; therefore, we investigated toxic effect, if any, of nHAP on plant growth promoting soil bacteria. Three types of plant growth promoting bacteria—nitrogen fixers (*Psuedomonas aeruginosas*), phosphate solubilizers and biofilm formers (*Bacillus subtilis*)—were exposed to nHAP. In addition, toxic-effect on commonly found gram positive and negative bacterial strains - opportunistic pathogen (*Acinetobacter baumannii*, gram negative) and non-pathogenic (*Escherichia coli*, gram negative and *Staphylococcus aureus*, gram positive) was also determined.

## Results and Discussion

The nanoparticles synthesized here are predominantly of HAP, and thus are referred to as nHAP hereafter. To distinguish between the biologically synthesized nHAP products made from different initial phosphorus concentrations (2%, 5%, 10% and 20%), 2P, 5P, 10P and 20P respectively are suffixed to nHAP. In order to differentiate the other forms of nHAP, suffixes Chem, Sigma and SRL are used to indicate nHAP obtained from chemical synthesis in-house, and from commercial vendors Sigma Aldrich and SRL respectively.

### Mechanism of synthesis of nHAP

It has been previously investigated that phosphorus solubilization can be achieved via growth of micro-organisms over various phosphate sources^30,34^. Previous studies have revealed the underlying mechanism of solubilization of insoluble P by microbial populations^35,36^. The principal mechanism involves production of mineral dissolving organic acids, siderophores, protons, hydroxyl ions and CO_2_. The organic acids are produced in the periplasmic space by the direct oxidation pathway. Secretion of these organic acids is accompanied by a drop in pH that results in the acidification of the microbial cells and the surroundings, hence, P ions are released by substitution of H^+^. Surprisingly, Asea *et al*. (1988) discovered that no correlation exists between the pH and the amount of P solubilization^37^. In another study, Illmer and Schinner (1995) proposed the theory of acidification by H^+^. They explained that for cationic assimilation, there is release of H^+^ ions making the overall solution acidic. For example, assimilation of NH_4_^+^ together with H^+^ secretion brings about P solubilization^35^. An alternative mechanism to organic acid production for solubilization of mineral phosphates is the release of H^+^ to the outer surface in exchange for cation uptake or with the help of H^+^ translocation ATPase.

**Figure 1:**
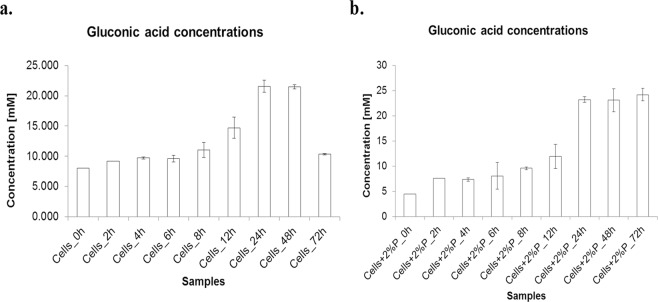
Gluconic acid concentrations at different time points for: (**a**). bacteria without P-source and (**b**). bacteria with P source.

**Figure 2:**
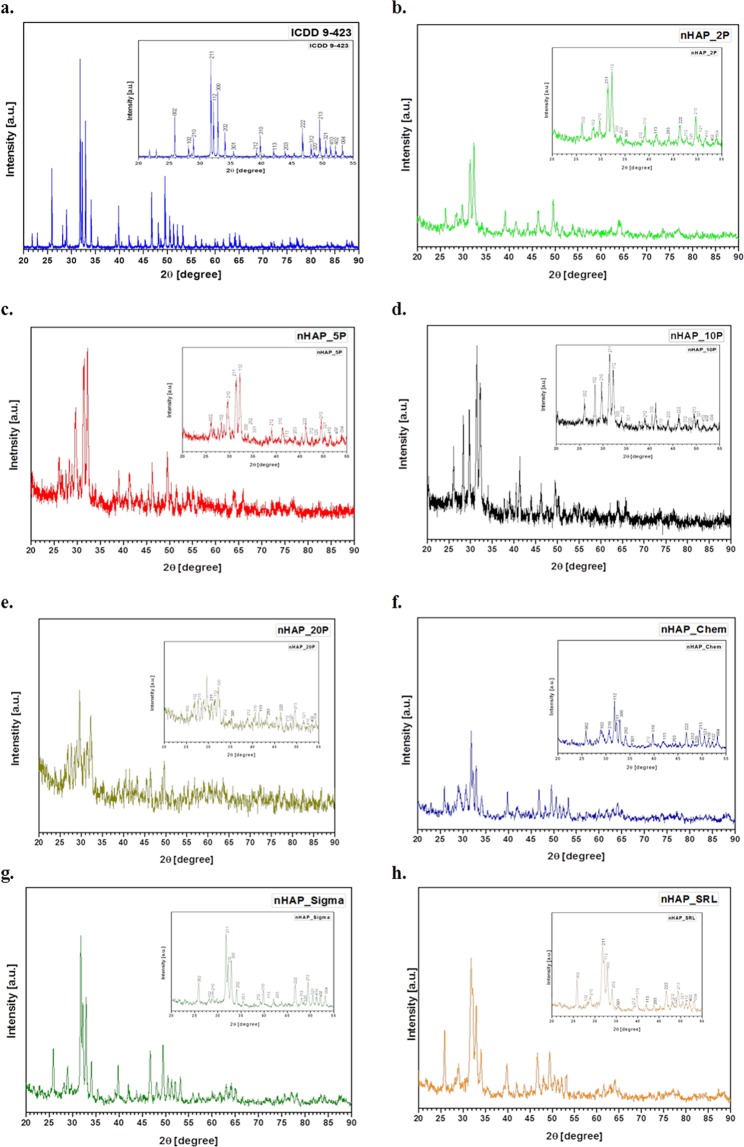
XRD diffractogram for different samples of hydroxyapatite. The miller indices corresponding to the signature hydroxyapatite phase peaks are depicted in the inset figure for each sample. (**a**) Shows the standard hydroxyapatite diffractogram from ICDD database with identity ICDD 9-423. (**b–h**) Show the diffractogram for nHAP_2P, nHAP_5P, nHAP_10P, nHAP_20P, nHAP_Chem, nHAP_Sigma and nHAP_SRL respectively.

We carried out HPLC based organic acid estimation to see correlation between release of particular organic acid/s (such as acetic acid, citric acid, gluconic acid, maleic acid, oxalic acid, pyruvic acid, succinic acid and tartaric acid) and P solubilization. Amongst various organic acids, gluconic acid showed significant correlation with P solubilization, whereas maleic and oxalic acids were not found in the reaction profile (SI, Fig. 2). It has been previously reported that gluconic acid is the most frequent agent of mineral phosphate solubilization in microorganisms. It chelates the cations bound to phosphate and thus makes the phosphate available to plants. Gram-negative bacteria solubilize mineral phosphate by direct oxidation of glucose to gluconic acid^7,30,32,38^. Pyrroloquinoline quinone (PQQ) acts as a redox cofactor in glucose dehydrogenases (GDH) resulting in phosphate solubilization^32,33,38^. The *pqq* gene cloning from an indigenous strain of *B. licheniformis* and heterologous expression in *E. coli* resulted in gluconic acid production and mediated phosphate solubilization activity^33^. In our experiments, we found a time dependent release of different organic acids (SI, Fig. 2). However, a direct correlation between increased acid concentrations when P source is added along with the feed can be seen only in the case of gluconic acid. It was observed that gluconic acid concentration increased, though not in an absolute linear manner, with time for the bacteria grown at 37 °C, 180 rpm (Fig. 1a). It is mentioned in literature that the degradation of gluconic acid happens under ambient conditions after 48 h, which is in agreement with observation made in our study (value drops from ~21 mM to ~10 mM, Table 1)^39^. However, we did not observe decrease in the concentration of gluconic acid even after 48 hours, when *B. licheniformis* was fed with P source. The concentration rather remained equivalent (~23–24 mM, Table 2) to that obtained at 24 hours (Table 2, Fig. 1b). This could be due to sustained release of gluconic acid by bacterium in the presence of P source^30^. Interestingly, presence of gluconic acid at 0 h was also seen in our experiment. This can be justified from the fact that the bacteria also produces some amount of gluconic acid constantly, which is independent of feed^32,39^, and is thus detected even at 0 h. Further, increased production of gluconic acid was seen up till 48 h when a P-source feed to bacteria was provided. No such correlation could be established for any other organic acid. Our results, therefore, emphasized a potential role of gluconic acid produced by *B. licheniformis* in P solubilization.

**Table 1:** Gluconic acid concentrations at different time points for bacteria without P source.

Samples	concentration[mM]	standard deviation
Cells_0h	8.050	0.181
Cells_2h	9.199	0.577
Cells_4h	9.719	1.229
Cells_6h	9.598	1.764
Cells_8h	11.036	0.999
Cells_12h	14.713	0.338
Cells_24h	21.557	0.121
Cells_48h	21.472	0.201
Cells_72h	10.323	1.056

**Table 2:** Gluconic acid concentrations at different time points for bacteria with P source.

Samples	concentration [mM]	standard deviation
Cells + 2%P_0h	4.487	0.348
Cells + 2%P_2h	7.597	2.645
Cells + 2%P_4h	7.335	0.278
Cells + 2%P_6h	8.070	2.399
Cells + 2%P_8h	9.587	0.569
Cells + 2%P_12h	11.900	2.275
Cells + 2%P_24h	23.221	1.242
Cells + 2%P_48h	23.111	0.478
Cells + 2%P_72h	24.200	0.950

The solubilized P then combined with Ca obtained from CaCl_2_ salt. The resultant mixture underwent gelation for 24 h. The nucleation and formation of nanoparticles thus could be explained by LaMer mechanism^40^.

The retention time and the concentration values of various organic acids are provided in the Supplementary Information (SI Figs 1, 2).

### Response of cell growth due to change in P concentration during bioconversion of substrate to nHAP

A microkinetic study on the bioconversion of nanohydroxyapatite was carried out in the batch mode. Monoculture of *B. licheniformis* was used as biocatalysts. In our study, biosynthetic conversion of nHAP, did not strictly follow an enzymatic process. The global conversion equation for this complex biotic-abiotic process followed the Monod’s substrate uninhibited model equation. Although a considerable quantum of work has been carried out on the microkinetics of the bioprocesses involving remediation of heavy metal ions from wastes, there is enough scope to investigate such a process in biological synthesis of nanoparticles leading to the evaluation of global reaction rate in terms of cell growth. Such kinetic study would establish the kinetic parameters for the bioreactor for upscaling of the process.

SI Fig. 3(a) represents the change in biomass during P-solubilization with respect to the untreated control bacteria. In the next step the intrinsic kinetic parameters μ_max_ and K_S_ were evaluated. It was done by reforming Eq. 1, which was followed by estimating the parameters from the intercepts of the axes in SI Fig. 3(b).

1 $$\mu =\frac{\mu max\ast S}{Ks+S}$$

2 $$\frac{1}{\mu }=\frac{Ks}{\mu max}\ast \frac{1}{S}+\frac{1}{\mu max}$$

When considering the change in biomass with time, the slopes for bacterial growth with and without P source were compared. The slopes were analyzed with linear regression and correlated using R^2^ goodness of fit. By two-tailed test for 95% confidence interval, p values for bacterial growth with and without P source were found to be 0.028 and 0.04 respectively. The deviations between the two was found to be statistically significant for p < 0.05, which suggested that the two growth patterns were different. For control set of experiments, K_s_ = 0.48 and μ_max_ = 0.02 was obtained in comparison to the treatment with P source, K_s_ = 1.38 and μ_max_ = 0.017. The increased Ks and μ_max_ values indicated the increase in bacterial biomass at a rate dependent on P source. A further correlation between the increases in bacterial biomass with the rise in gluconic acid secretion in the treated set of experiments was observed.

Bioconversion of soluble P, when viewed as a single step, was not only a fermentative process. However, when the global rate involving both complex biotic-abiotic processes were considered, the P solubilization was found to follow the Monod substrate uninhibited model equation from 0 h to 72 h in our study. Based on the experimental data, we proposed for the following global rate equation for P solubilization by *B. licheniformis, which may be* applied for estimating space time and reactor volume for up-scale of synthesis at fermenter scale in future:

3 $$\mu =\frac{0.017\ast S}{1.38+\,S}$$

### Measurement of hydrodynamic size and zeta potentials of nHAP

The hydrodynamic diameter (HDD) and the zeta potential (ZP) were measured using a zeta-sizer and the results for each of the different samples of nHAP are reported in Table 3. The size and zeta potential varied between each of the nanomaterial samples. The biologically synthesized nHAP samples presented a smaller hydrodynamic size in comparison to the chemically synthesized particles. The biologically synthesized nHAP (nHAP_Bio) exhibited a size range of 269.8–419.1 nm, which was 2.8–1.8, times smaller than chemically synthesized particles (nHAP_Chem, HDD- 756.2 nm).

**Table 3:** Hydrodynamic diameter, zeta potential, polydispersity index and TEM sizes of different samples of nanohydroxyapatite.

Sample	Hydrodynamic diameter [d.nm]	Polydispersity index [PdI]	Zeta potential [mV]	Size from TEM [nm]
nHAP_2P	325.8 ± 37.1	0.268 ± 0.233	−31.3 ± 3.5	35.74 ± 11.65
nHAP_5P	269.8 ± 17.3	0.207 ± 0.014	−37.8 ± 0.5	L = 126.66 ± 31.6
				W = 39.6 ± 8.56
nHAP_10P	338.5 ± 11.5	0.486 ± 0.113	−51.9 ± 0.3	L = 129.7 ± 21.99
				W = 48.42 ± 1.74
nHAP_20P	419.1 ± 12.9	0.455 ± 0.026	−44.8 ± 0.9	L = 98.92 ± 10.16
				W = 36.6 ± 8.49
nHAP_Chem	756.2 ± 28.8	0.531 ± 0.094	−45.2 ± 1.7	L = 83.92 ± 17.98
				W = 26.85 ± 3.74
nHAP_Sigma	874.3 ± 51.5	0.299 ± 0.036	−10.3 ± 0.3	33.9 ± 8.6
nHAP_SRL	892.8 ± 21.1	0.267 ± 0.018	−9.7 ± 0.9	L = 64.64 ± 4.33
				W = 14.01 ± 1.26

Dispersal medium – DI water, sample concentration – 1 mg/mL.

The ionic end groups at the interfaces of particles prevent Van der Waals attraction, which causes coating of surfaces, coagulation, aggregation, flocculation and coalescence of dispersions. The higher the electrostatic repulsion between particles, the lower is the probability for collisions of Van der Waals type. The values for ZP were found in the range of −31.3 to −51.9 mV for nHAP_Bio and −45.2 and −10.3 for nHAP_Chem. High ZP values from the electrical double layer formed in aqueous state around the particles corroborated aqueous stability of synthesized nanoparticles in agreement to the classical DLVO theory^41^.

The low range of polydispersity index (PdI) suggests for the homogeneity in size of the samples. A PdI in the range of 0.268–0.455 for nHAP_Bio and 0.531 for nHAP_Chem was observed.

### Powder characterization of nanoparticles formed using X-ray diffraction (XRD)

A typical powder XRD pattern with the prime Miller (h k l) indices [(002), (211), (300), (202), (130), (002), (222) and (213)] of nHAP has been presented in Fig. 2(a–h). The inspection of the XRD patterns of the nHAP_Bio (Fig. 2b–e), nHAP_Chem (Fig. 2f), products from Sigma (Fig. 2g) and, SRL (Fig. 2h) revealed the presence of prominent crystalline nanometer sized HAP phases, which were consistent with the phases listed in the ICDD database id as 09432 (Fig. 2a). In addition, with different concentrations of P used in synthesis of nHAP, presence of phases other than pure HAP could be seen in the background of the major phase for nHAP (Fig. 2). The additional phases could be of α and β tri-calcium phosphate, tetra-calcium phosphate and/or calcium oxide, which were of amorphous nature. The proportion of such amorphous phases increased with increase in P concentration during synthesis. Our data was also in agreement with the peak positions for HAP previously described in the literature^42^. The peak shifts from the standard HAP spectra could be correlated with the Ca/P ratios. The samples (nHAP_Chem, nHAP_Sigma, nHAP_SRL and nHAP_2P) having the classical Ca/P ratio of 1.677 showed phases similar to standard HAP^43^. nHAP_5P, nHAP_10P and nHAP_20P (Fig. 2c–e) showed additional phases and different shapes (as shown by SEM and TEM micrographs) when compared to standard HAP^42^. In case of the Ca:P ratio < 1.5, phases for di-calcium and tri-calcium phosphates could have infiltrated HAP phases whereas, for samples with Ca:P > 1.677, calcium oxides and calcium hydroxide phases could be present. The peak at 31.8° represented the crystalline HAP form. The varying intensity presented the preferential crystallographic orientation and thus the difference in lattice structure, ultimately resulting in differently shaped particles. The (2 1 1) reflection peak from the XRD pattern was used to calculate the nHAP crystallite size in this study from the Debye-Scherrer equation^44^ and was estimated to have a mean value of 30 ± 5 nm, which was in agreement to the size obtained via electron microscopy.

### Fourier transformed infra-red (FTIR) spectroscopy of nHAP

**Figure 3:**
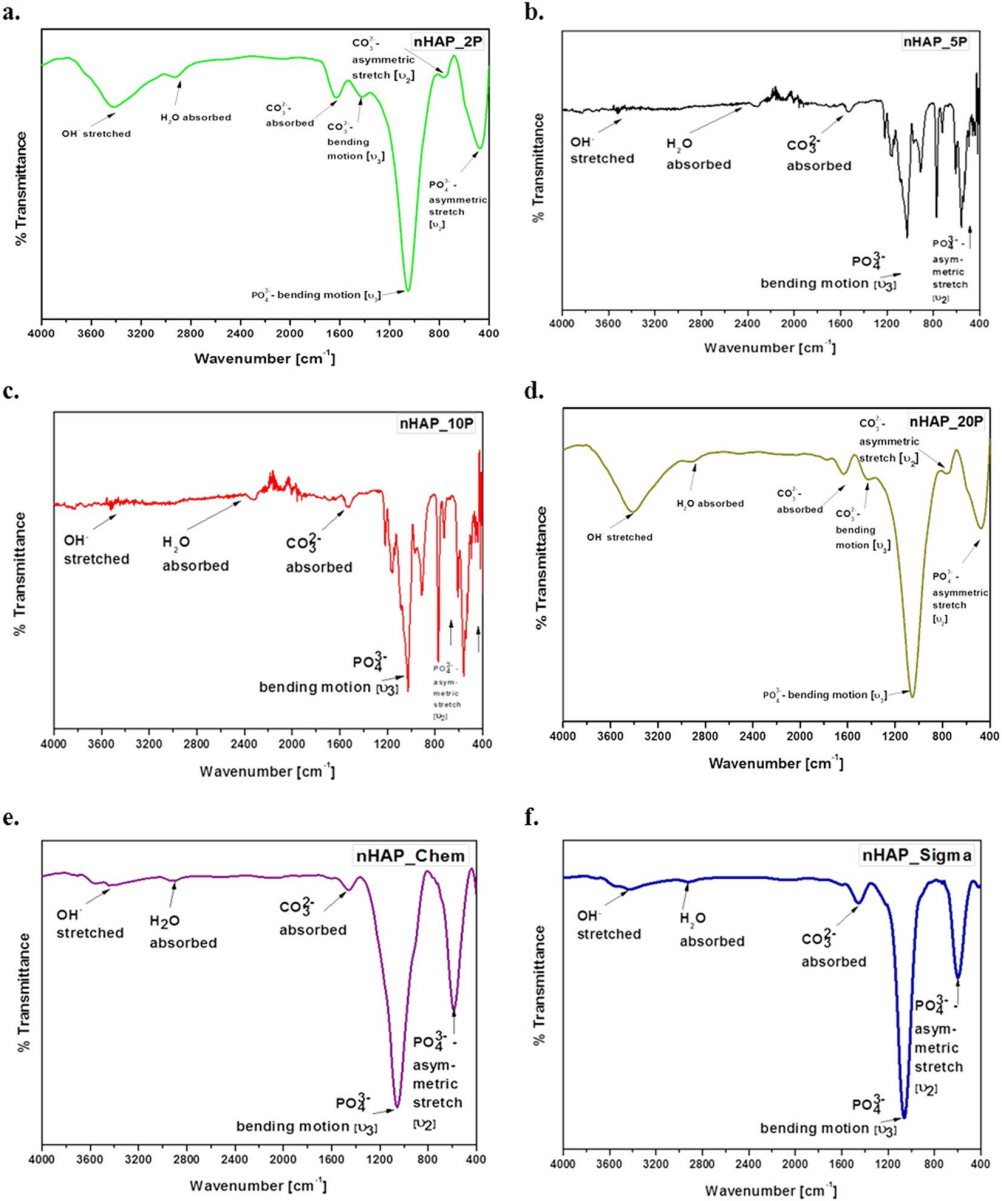
FTIR spectra (4000–400 cm^−1^) for different samples of nano-hydroxyapatite showing bond movements due to OH^−^ stretching, H_2_O absorption, CO_3_^2−^: absorption, bending and stretching; and PO_4_^3−^: bending and stretching. Descriptors: (**a**) nHAP_2P, (**b**). nHAP_5P, (**c**) nHAP_10P, (**d**) nHAP_20P, (**e**) nHAP_Chem and (**f**) nHAP_Sigm.

The different methods to synthesize HAP may lead to different vibrational modes in the detected PO_4_^3−^ groups^45^. These differently vibrated groups may provoke characteristic changes in the lattice parameters, crystallinity, crystal symmetry, thermal stability, morphology, and solubility, physical, chemical and biological characteristics^44^. Theoretically, the most characteristic chemical groups in the FTIR spectrum of synthesized HAP were PO_4_^3−^, OH^−^, CO_3_^2−^, as well as HPO_4_^2−^ that characterized the non-stoichiometric HAP^46^. PO_4_^3−^ group formed intensive IR absorption bands at 560 cm^−1^, 600 cm^−1^, and at 1000–1100 cm^−1^. The ion stretching vibration around 3568 cm^−1^ confirmed the presence of a hydroxyl group. Adsorbed water band was relatively wide, with an explicit peak around 2800 cm^−1^. Likewise, the other stretching vibrations for carbonyl and phosphate groups were also observed as reported earlier. CO_3_^2−^ group formed intensive peaks between 1460 and 1530 cm^−1^. Absorption peaks of chemical bonds of the synthesized HAP spectrum have been summarized in Fig. 3(a–f). The functional groups of the nHAP powder predicted from FTIR spectra analysis were compared with the results of Meejoo *et al*.^47^, for the confirmation. Absence of peaks corresponding to HPO_4_^2−^ confirmed the intact stoichiometry of the synthesized hydroxyapatite. A CO_3_^2−^ υ2 asymmetric stretch could be observed in the IR spectra of biologically synthesized nHAP (nHAP_2P, nHAP_5P, nHAP_10P and nHAP_20P; Fig. 3a–d). This has been suggested to signify substitution of phosphate ion by CO_3_^2−^ group resulting in formation of B-type HAP^47^. Such B-type HAP formation has been reported as the most commonly found biological form of HAP^47^. The sharp bands in the range of 1050–1100 cm^−1^ arise from υ3 bending motion of PO_4_^3−^ group whereas less transmitted intense bands from 400–600 cm^−1^ are due to υ2 asymmetric stretch of PO_4_^3−^.

### Scanning electron microscopy (SEM) of nHAP

**Figure 4:**
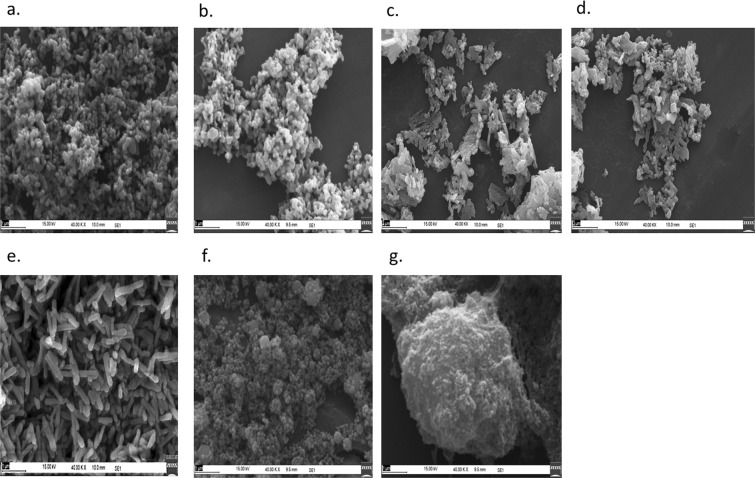
Scanning electron micrographs of different samples of nano-hydroxyapatite at 1 μm scale bar, 15 kV voltage and 40k X magnification. (**a**) nHAP_2P, (**b**) nHAP_5P, (**c**) nHAP_10P, (**d**) nHAP_20P, (**e**) nHAP_Chem, (**f**) nHAP_Sigma, (**g**) nHAP_SRL.

SEM images of biologically and chemically synthesized and commercially available nHAP are presented in Fig. 4(a–g). The different nHAP were composed of either spherical (Fig. 4f, nHAP-Sigma), or platelet (Fig. 4a, nHAP_2P) or rod-shaped particles (Fig. 4b–e,g: nHAP_5P, 10P, 20P, Chem and SRL). Fairly consistent size and shape distribution in agreement with the XRD and TEM analysis was observed. In comparison with the commercial analogues – the biologically synthesized nHAP_2P showed similar morphology and distribution with that of nHAP_Sigma. With the increase in the source P concentration, the particles showed more rod-shaped morphology which was comparable to nHAP_Chem and nHAP_SRL. There was no evidence of hollowness or continuous empty space inside the nHAP particles. In agreement with our observations, the previously reported predominant forms of nHAP included spherical^48^, platelet^49^ and rod/needle shaped^50^ particles. The porosity and hollowness of the particles have been shown to be dependent on route of synthesis and sintering/calcination temperature^51^.

### Transmission electron microscopy (TEM) of nHAP

**Figure 5:**
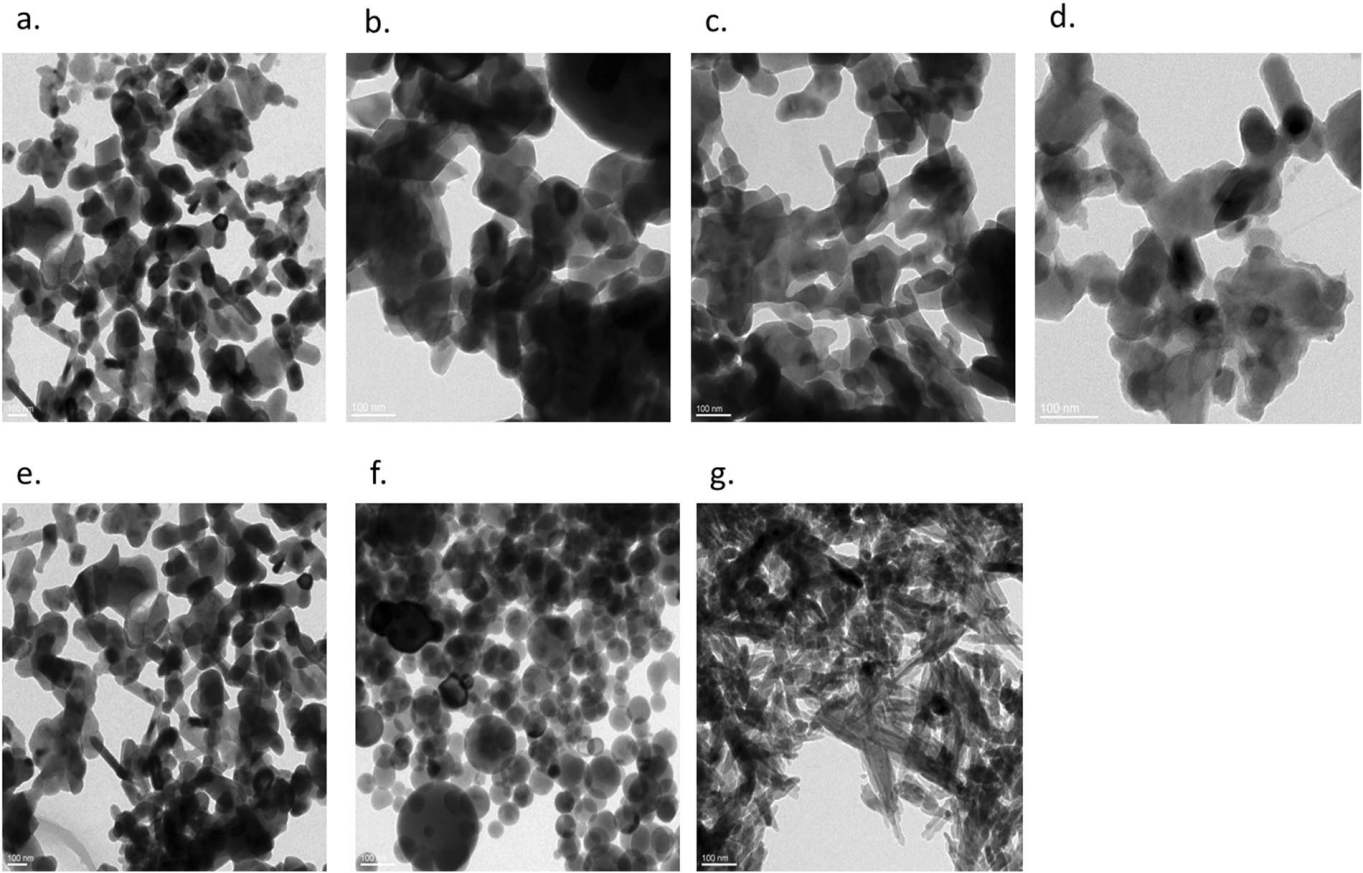
Transmission electron micrographs of different samples of nano-hydroxyapatite at 100 nm scale bar. (**a**) nHAP_2P, (**b**) nHAP_5P, (**c**) nHAP_10P, (**d**) nHAP_20P, (**e**) nHAP_Chem, (**f**) nHAP_Sigma, (**g**) nHAP_SRL.

Size and morphology of different nHAP samples were studied by TEM (Fig. 5). The particles were mainly of spherical, platelet and rod/needle shaped morphology and were in the range of 20–50 nm in different dimensions. Both rod and platelet shaped particles were observed in the biologically synthesized samples. The particles acquired rod/needle shaped morphology at higher P-concentrations during the biosynthesis of nHAP. The overviews of micrograph of nHAP-Bio were comparable with the commercially available and chemically synthesized controls from Sigma Aldrich and SRL Pvt. Ltd. – nHAP_Sigma and nHAP_SRL respectively. nHAP_2P was nearly spherical/platelet shaped with size 35.74 ± 11.65 nm (Fig. 5a). nHAP_5P (Fig. 5b), nHAP_10P (Fig. 5c) and nHAP_20P (Fig. 5d) are rod shaped particles with lengths × width as 126.66 ± 31.6 nm × 39.6 ± 8.56 nm, 129.7 ± 21.99 nm × 48.42 ± 1.74 nm and 98.92 ± 10.16 nm × 36.6 ± 8.49 nm respectively. The particles of the in house developed chemically synthesized nano-hydroxyapatite (nHAP_Chem) were rod shaped with dimensions as 83.92 ± 17.98 nm (length) and 26.85 ± 3.74 nm (width). Commercially available analogues were spherical with a size of 33.9 ± 8.6 nm for nHAP_Sigma and rod/needle shaped with length as 64.64 ± 4.33 and width as 14.01 ± 1.26 for nHAP_SRL. The sizes were in agreement with the size obtained through FWHM (full width of half maxima) analysis of the XRD diffractogram and the morphology was consistent with SEM analysis.

### Elemental characterization: P and Ca analysis of synthesized nanoparticles

**Figure 6:**
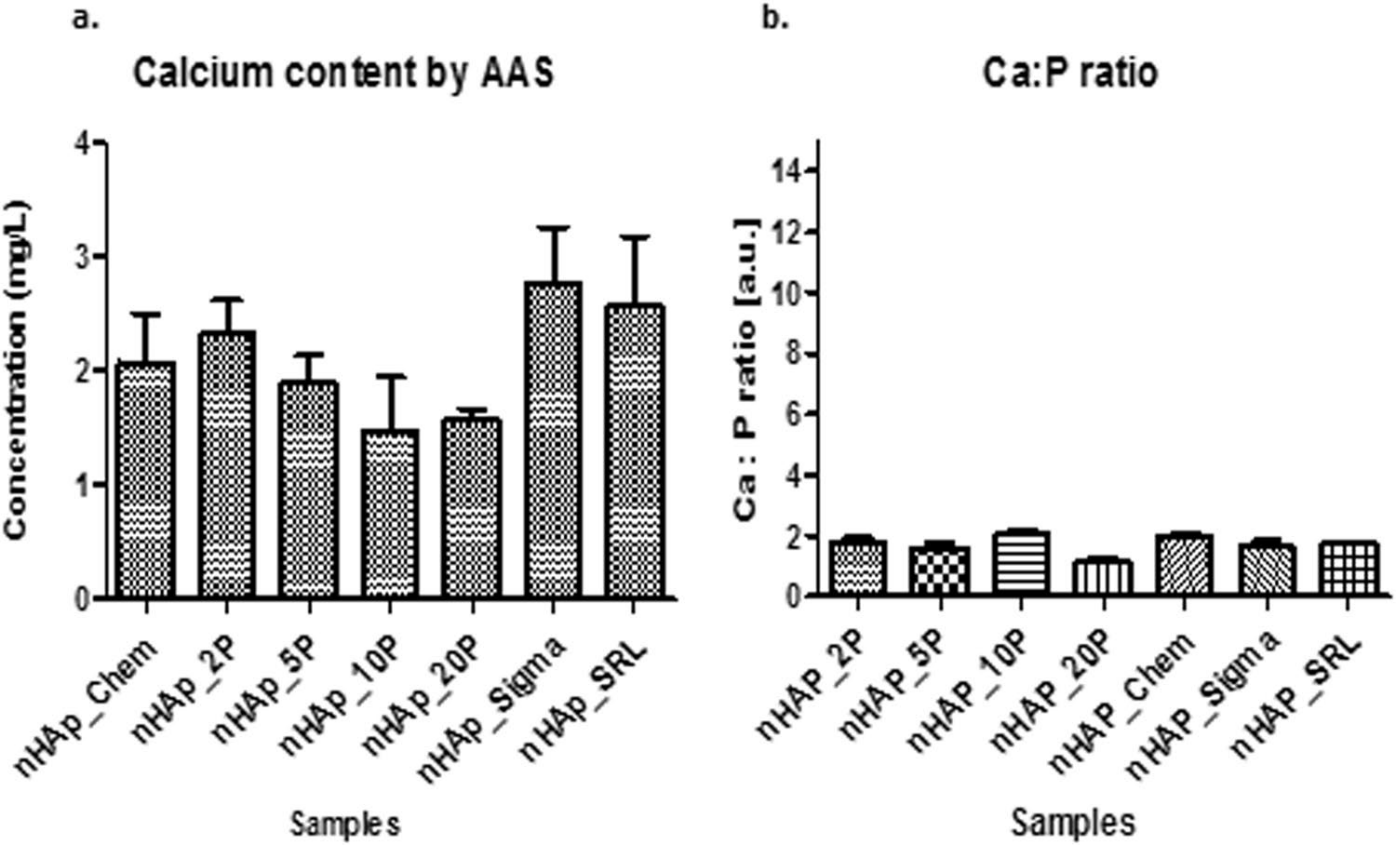
Elemental composition and Ca/P ratio for different samples of nanohydroxyapatite. (**a**) Shows the Calcium content in each sample of nanohydroxyapatite. (**b**) Shows the Ca/P for the different samples of nanohydroxyapatite.

Calcium content was estimated using AAS (Fig. 6a), whereas the phosphorus content was analytically estimated using an end-point colorimetric method. The elemental characterization was influenced by both P concentration and the sample type.

As reported in the literature, the classical hydroxyapatite ratio is 1.677^52^. Ca/P ratio for nHAP_Chem, nHAP_2P, nHAP_Sigma and nHAP_SRL fall in range between1.6–1.7, which suggested chemical similarity to hydroxyapatite as depicted in Fig. 6b. The other variations could possibly be due to the presence of other additional phases of calcium phosphate such as α and β tricalcium phosphate and tetracalcium phosphate. The data could be further explained with EDX and XRD analysis to conclude on presence of other elemental contamination and various phases of calcium phosphate other than HAP. The calculated Ca/P ratio was found to be in good agreement with those reported previously for HAP.

### Energy dispersive X-ray spectroscopy (EDX) of synthesized particles

**Figure 7:**
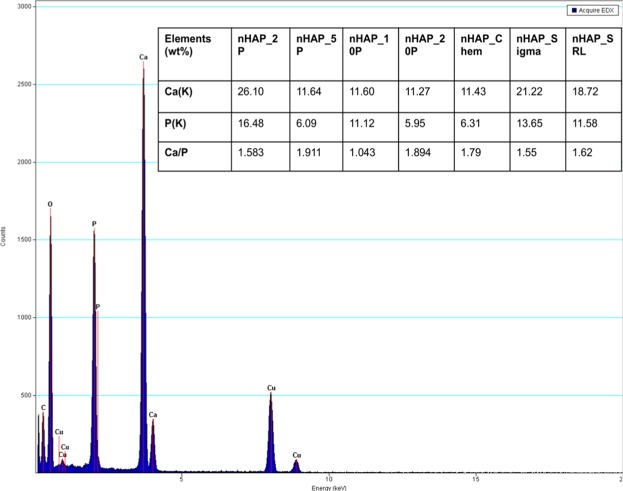
Representative EDX data and elemental compositions in atomic percentage (inset table) for samples of nanohydroxyapatite.

For chemical constituent identification of nHAP the atomic Ca/P ratio was monitored by EDX. When applying EDX to the samples, the mean Ca/P ratios were 1.583, 1.911, 1.043, 1.894, 1.79, 1.55 and 1.62 for nHAP_2P, nHAP_5P, nHAP_10P, nHAP_20P, nHAP_Chem, nHAP_Sigma and nHAP_SRL respectively. A Ca/P ratio <1.5 indicates biphasic mixtures composed of a calcium phosphate apatite and CaHPO_4_ whereas that of >1.667 contains calcium phosphate apatite and Ca(OH)_2_^53^. Elemental analyses revealed similar mean Ca/P ratios as that of HAP for nHAP_2P and concomitant to Ca/P ratio reported for HAP in the literature. It could be concluded that the EDX measurements were good approximations for the elemental composition of nHAP. A representative image for EDX is depicted in Fig. 7.

### Zone of inhibition study in PGPR bacteria against synthesized nHAP

Zone of inhibition study was done to evaluate effects of the synthesized nHAP on different soil bacteria. The results, as depicted in SI, Fig. 4 confirmed absence of any bactericidal activity of nHAP against any of the strains of soil bacteria and plant-growth-promoting bacteria included in this study. The broad spectrum antibiotic, gentamycin, which was used as a positive control, exhibited effective bactericidal effects^54^.

## Methods

The study involved biosynthesis and characterization of nHAP. For all comparative analyses, chemically synthesized and commercially available nHAP were used for comparison. Materials, techniques and details about samples preparation employed for characterization are described in the following section. Ultrahigh-pure water (minimum resistivity: 18.2 MΩ cm), that hereinafter will be referred as de-ionized (DI) water, produced by a MilliQ water purifier system (Millipore, Massachusetts, USA) was used throughout in the experimental assays.

### Materials

Nutrient broth, nutrient agar (HiMedia, Mumbai, India); K_2_HPO_4_, CaCl_2_, NaOH, ammonium persulfate, nanohydroxyapatite (Sigma-Aldrich, Missouri, USA); gluconic acid, sulphuric acid, nitric acid, hydrochloric acid (Merck Millipore, Massachusetts, USA); ammonium molybdate, antimony potassium, ascorbic acid (Fischer Scientific, Massachusetts, USA), nanohydroxyapatite Sisco Research Laboratories Pvt. Ltd. (Mumbai, Maharashtra, India).

### Biological synthesis

In this study the biological synthesis of nHAP was done using a strain of *Bacillus licheniformis* (received from the germplasm collection at TDNBC, TERI, Gwal Pahadi, Gurugram, India). To prepare the bacterial inoculum (pre-culture), aseptic nutrient broth (NB) was prepared. 10 mL of heat sterilized (20 min, 15 psi, 121 ± 1 °C) NB medium was aseptically inoculated with a single bacterial colony followed by incubation at 37 °C and 200 rpm in a shaker incubator (LabTherm, LT-X, Kuhner, Switzerland) for 12 hours (h). To prepare the bacterial culture for the synthesis of nHAP, 100 mL of NB was inoculated with 1% *v/v* of pre- culture and different concentrations of K_2_HPO_4_ [2%, 5%, 10% and 20% (*w/v*)] were added to four individual sets of experiments and incubated at 37 °C, 200 rpm for 24 h. After 24 h, the fermented broth was centrifuged at 10,000 rpm, 25 °C for 15 minutes (min) (5804 R, Rotor: F-34-6-38, Eppendorf, Germany). Supernatants were collected for each set followed by analysis for P concentration and sufficient amount of CaCl_2_ was added so that the final ratio of Ca/P was maintained at 5:3. The reaction mix (supernatants + CaCl_2_) was incubated at 37 °C, 200 rpm for 24 h after which the reaction mix was centrifuged at 10,000 rpm, 25 °C for 15 min and pellets were recovered. The pellets were washed thrice with double-distilled water (d.dH_2_O) and were allowed to dry. Dried pellets were then autoclaved (20 min, 15 psi, 121 ± 1 °C) followed by calcination at 500 °C for 6 h with 10 °C min^−1^ ramp rate in muffle furnace (SHI-205, Shivam Instruments, Delhi, India) and the obtained product was used for further analysis.

Agar plate test was carried out to rule out the possibility of microbial contamination in the preparation of nHAP. Briefly, nHAP samples along with controls (positive control: bacterial culture at 10^−6^, 10^−7^ and 10^−8^ dilutions and negative control: DI water) were spread on the surface of nutrient agar and growth of bacterial colonies was monitored up to 72 h of incubation.

### Chemical synthesis

To prepare the control samples for comparative study with biologically synthesized nHAP, chemical synthesis was carried out under pH static conditions by following the protocol for wet chemical synthesis through sol-gel technique^55,56^. Briefly, K_2_HPO_4_ and CaCl_2_ were mixed in d.dH_2_O and a Ca/P ratio of 5:3 was maintained. The pH of the solution was maintained at 10 by adding 0.1 M sodium hydroxide. The mixture was continuously stirred for 10 min at ambient temperature followed by heating in a water bath (Sun Scientific, Delhi, India) at 60 °C for 1 h. A white transparent gel was obtained. The gel was aged for 4 h at ambient temperature followed by drying at 80 °C for 24 h in a hot air oven (Thermocenter, SalvisLab, Rotkreuz, Switzerland). The dried gels were calcined at 10 °C min^−1^ ramp rate to 500 °C in a muffle furnace, and then air cooled to ambient temperature. The sintered product was crushed using an agate mortar and pestle to obtain resultant nano-powder.

### Mechanism of synthesis

The mechanism of synthesis of nHAP was investigated by quantitative analysis of various organic acids (acetic acid, citric acid, gluconic acid, maleic acid, oxalic acid, pyruvic acid, succinic acid and tartaric acid) produced during the synthesis. The reaction mix having bacterial culture and K_2_HPO_4_ was analyzed at different time intervals (0 h, 2 h, 4 h, 6 h, 12 h, 24 h, 48 h and 72 h) and various organic acids were quantified. Standards of different organic acids were used to prepare the calibration curve. The analysis was done by high performance liquid chromatography (HPLC) (Shimadzu, Kyoto, Japan) using an organic acid column (Allure organic acids 5 μm, 25 × 4.6 mm, Restek, Pennsylvania, USA) and 0.01 M sulphuric acid as mobile phase. To prepare for HPLC analysis, samples were collected after completion of pre-determined reaction time and centrifuged at 14,000 rpm for 30 minutes and at 4 °C. The supernatants were then filtered through 0.22 μ filters (MDI, Pennsylvania, USA) and subjected to auto-sampler (SIL-20A, Shimadzu, Kyoto, Japan). The concentration of each organic acid was determined from the respective standard curves.

### Cell growth kinetics

#### Batch experiments

To determine growth kinetics of *B. licheniformis* with respect to P concentrations in culture medium due to added substrate, batch experiments were conducted in Erlenmeyer flasks with constant shaking. For each run the working hold-up volume was maintained at 100 mL containing 10% of inoculum. A constant temperature of 37 °C was maintained. Samples were withdrawn at an interval of 1 h with total time span of 72 h.

#### Determination of biomass concentration

Concentration of bacterial mass in the reaction broth of batch type experiments under steady state was determined both spectrophotometrically and by dry weight method. In this method, cell concentration of bacterial suspension withdrawn at different time intervals was spectrophotometrically determined at 600 nm. For dry weight method, 2 ml nutrient broth enriched with bacterial strain was taken in pre-weighed MCT. It was then centrifuged at 10,000 rpm for 15 min. The separated biomass was washed thrice with de-ionized water and dried at 80 °C for 24 h. The exact weight of the bacterial mass was determined by subtracting the empty weight of the MCT from that of the respective MCT containing dry bacterial mass.

#### Theoretical analysis

As mentioned earlier, in this study, formation of nanohydroxyapatite is not a metabolic process in its strict sense, since the microorganism produces high amount of gluconic acid for P solubilization. Also, the process is followed by mimicking of classical sol-gel approach and thus cannot be considered as a direct enzymatic process at reaction scale. However, a close theoretical inspection for the reaction mechanism reveals involvement of *pqq* gene cluster that is responsible for production of gluconic acid during biotic microbial action on substrate through the enzymatic process. Microkinetics of the formation of nHAP can, thus, be logically considered as a particular step in the overall intercellular enzymatic process. Accordingly, the production of nHAP may be regarded as the effect of a chain of fermentative reactions. In the present investigation, evaluation of the global reaction rate kinetics of the production of nanohydroxyapatite was carried out using batch process with the following considerations^57^:

(i) The reaction was assumed to be ideal in nature. The small reactor volume (500 mL) and high stirrer speed (200 rpm) was considered to be adequate for this assumption.
(ii) All observations were made when the reaction attained steady state condition.
(iii) The reaction was considered as a constant density system since only liquid phase reaction was involved.
(iv) The yield coefficients were computed from the constant slope of the plot of cell concentration vs. substrate concentration. These data were obtained from cell growth study in the batch mode of operation.
(v) The cell yield coefficient was assumed to be constant during the subsequent mathematical analysis. This assumption was reasonably valid during the exponential phase of the cell growth.

Monod’s classical substrate uninhibited model equation is still an important tool developing a suitable global model equation for a product obtained through the fermentative process. Biosynthesis of nanohydroxyapatite in the overall metabolic pathway is rather complex. However, based on the principle that if a simple rate equation can describe the reaction engineering behaviour of a complex system, it was decided to check the suitability of Monod’s substrate uninhibited model equation in the present case of the complex bioconversion process of nanohydroxyapatite synthesis. Levenspiel^58^ has presented an excellent analysis of chemostat performance when the system follows Monod’s substrate uninhibition model equation. In such a case, the cell growth rate is given as –

4 $$\mu =\frac{\mu max\ast S}{Ks+S}$$

### Physicochemical characterization

Hydrodynamic size, polydispersity index and zeta potential of the synthesized particles were evaluated using Zetasizer Nano-ZS (Malvern instruments, UK). Further morphological studies of samples were done using SEM (EVO, MA10, Carl Zeiss, Germany) operated at 15 kV and TEM (Tecnai G2 30-U twin microscope, FEI, Massachusetts, USA) at voltage of 200 kV. EDX coupled with TEM was also monitored for the elemental distribution. For functional group analysis using FTIR, the samples were prepared by KBr disk method^59^. Analysis was performed on Thermofisher FTIR spectrometer with FIR attachment (Nicolet iS50 FTIR Tri-detector, Massachusetts, USA) at room temperature in the range of 4000–400 cm^−1^ with 100 scans per sample. The synthesized particles were also characterized by X-ray diffraction (XRD). Powder XRD spectra were recorded at room temperature, using Bruker, D8 discover high resolution X-ray diffractometer (Bruker, Massachusetts, USA) with Cu Kα = 1.5406 Å, 3 kW as radiation source operating at 40 kV and 40 mA. The diffraction patterns were collected over a 2θ range from 20° to 90° with an incremental step size of 0.02° using flat plane geometry. The acquisition time was set at 2 seconds for each scan. The crystalline size of the particles in the powders was calculated using the Debye-Scherrer equation from the respective XRD patterns^44^.

### Elemental characterization: P and Ca analysis

P and Ca analysis was done to determine the Ca/P ratio for the synthesized nHAP. The detailed methodology is provided in the Supplementary Information. The Ca analysis was done using atomic absorption spectroscopy and P analysis was done using colorimetry assay by following the classical reaction between molybdate and phosphate groups to give a blue-purple color as end-point detection.

### Microbiological assays

#### Strains and their growth conditions

*Staphylococcus aureus* (MTCC 11949), *Escherichia coli* (MTCC 1610), *Pseudomonas aeruginosa* (NRRL B59191), *Acinetobacter baumannii* (MTCC 9869) and *Bacillus subtilis* (NEUB) were grown at 37 °C in freshly prepared nutrient broth prior to each experiment. Fresh inoculums were prepared by taking one colony in nutrient broth and simultaneously compared the turbidity with McFarland standard of absorbance 0.5 (9.8 × 10^8^ CFU/mL for *Staphylococcus aureus*, 5.2 × 10^7^ CFU/mL for *Escherichia coli*, 8.7 × 10^7^ CFU/mL for *Pseudomonas aeruginosa*, 1.2 × 10^8^ CFU/mL for *Acinetobacter baumannii*, and 5.8 × 10^7^ CFU/mL for *Bacillus subtilis*).

#### Zone of inhibition

Zone of inhibition was performed by agar well diffusion method on nutrient agar. The inoculums were prepared using fresh plate cultures of microbes as stated above. On solidified agar plates (150 cm diameter), wells with 6 mm diameter were cut out. 50 μL of the nanomaterials at concentrations 0, 1.5625, 3.125, 6.25, 12.5, 25, 50, 100, 200, 500 and 1000 μgmL^−1^ along with non-nano derivatives (Ca_3_PO_4_ for nanohydroxyapatite) were carefully placed into each well. The plates were first incubated at 4 °C for 15 min (to allow the proper settling of nanomaterial in wells) followed by incubation at 37 °C for 16 h. Anti-bacterial activity was determined by measuring the zone of inhibition with gentamycin (10 μg/mL) as a positive control. The zone of inhibition was measured using Antibiotic Zonescale PW096 (HiMedia Labs, India).

### Statistical analysis

All data are reported as mean ± standard deviation from the replicated results. The data from different experiments involving assessment of composition (AAS, EDX and chemical analysis), size (DLS and TEM), polydispersity index and zeta-potential were statistically analyzed using Graphpad prism and Origin Pro 8.

## Conclusions

In this study, we reported *Bacillus licheniformis* mediated biosynthesis of a nanostructured hydroxyapatite powder by using calcium and phosphorous precursors. The major mechanism underlying nanoparticle synthesis involved P solubilization by production of gluconic acid through the *pqq* gene cluster present in *B. licheniformis*. This solubilized P then combined with Ca in a sol-gel manner to yield nHAP. The biogenic nHAP exhibited physicochemical properties at par with chemically synthesized and commercially available hydroxyapatite nanoparticles. Evaluation of effect of biosynthesized nHAP on soil microbes with the futuristic aim of using the product as P nano-fertilizer showed no adverse impact on the growth of different gram-positive and gram-negative strains of soil bacteria. Results from the study provide wide scope of evaluation of the synthesized nHAP_2P for application in various fields.

## Supplementary information

Supplementary information
