# Supplementary material for: A new method for biological synthesis of agriculturally relevant nanohydroxyapatite with elucidated effects on soil bacteria
Source: Sci Rep. 2019 Oct 21;9:15083. doi: 10.1038/s41598-019-51514-0 (PMC6803707; doi:10.1038/s41598-019-51514-0)
Supplement: Supplementary file 1 — Supplementary information [file 41598_2019_51514_MOESM1_ESM.docx]

**SUPPLEMENTARY INFORMATION**

**A new method for biological synthesis of agriculturally relevant nanohydroxyapatite with elucidated effects on soil bacteria**

Ayushi Priyam^1, 2^, Ratul Kumar Das^1^, Aaron Schultz^2^, Pushplata Prasad Singh^1,2^*

Correspondence to [pushplata.singh@teri.res.in](mailto:pushplata.singh@teri.res.in)

^1^National-Centre of Excellence for Advanced Research in Agricultural Nanotechnology, TERI - Deakin Nanobiotechnology Centre, Sustainable Agriculture Division, The Energy and Resources Institute (TERI), DS Block, India Habitat Centre, Lodhi Road, New Delhi, 110003, India

^2^School of Life and Environmental Sciences, Deakin University, Geelong, Victoria, 3217, Australia.

**Methods -**

- 1. **Elemental characterization: P and Ca analysis**
     - 1. *Calcium analysis:* calcium analysis was conducted using atomic absorption spectroscopy (AAS). It is hypothesized that nano-conversion results in an increase in overall available calcium content. Presence of calcium in nanoparticle samples was quantified by atomic absorption spectrophotometry (iCE 3000 AA05123903 v1.30, ThermoFisher Scientific, USA). Analysis was carried out on nanoparticle samples prior to and after acid digestion. A 1000 ppm concentration was prepared for the samples by dissolving the appropriate amount in de-ionized water. For acid digestion, nanoparticle in powdered form (5 mg) from each sample was digested in 5 ml of a mixture of nitric acid and hydrochloric acid (in the ratio 1:3). The acid treated samples were then boiled gently at 95°C for 4 hours by heating in a water bath (Sun Scientific, Delhi, India). The digested samples were then diluted with de-ionized water to obtain a final working concentration of 5 ppm (dilution factor: 200). The standards of different calcium concentrations (i.e. 0.5, 1 and 2 ppm) were prepared (positive controls) from commercial standard solution (Loba Chemie, Mumbai, India). The standards and sample solutions were read against de-ionized water (negative control) as a blank solution. Flame analysis was carried out with N_2_O-C_2_H_2_ flame type and absorbance was measured at 422.7 nm. Prominently, phosphate can have an influence on number of calcium atoms in the flame capable of absorbing light. By using simple air – acetylene flame, phosphate does not dissociate, therefore its concentration remains pertinent resulting in decreased calcium absorption. N_2_O-C_2_H_2_ was chosen to avoid such chemical interferences. Final concentration of calcium for each sample was then displayed by the instrument in an automated manner. The experiment was conducted in triplicates.
       2. *Phosphorus analysis:* To analyze the basic elemental ratio of Ca/P, phosphorus estimation as described in the protocol 365.3 by EPA, USA was followed. The method follows a classical reaction between molybdate and phosphate groups to give a blue-purple color as end-point detection. The reaction is carried out in an acidic solution containing excess ascorbic acid (vitamin C) to prevent the complex from slowly oxidizing. Briefly, a 1000 ppm concentration was prepared for the samples by dissolving the appropriate amount in de-ionized (DI) water. 50 mL of samples were transferred into a 125 mL Erlenmeyer flask and 1 mL of 11N sulfuric acid was added. To this, 0.4 g ammonium persulfate was added, mixed and boiled gently for approximately 30-40 minutes or until a final volume of about 10 mL is reached. The reaction mix was cooled and diluted to approximately 50 mL. Next, 4 mL of ammonium molybdate (0.8% w/v) and antimony potassium tartrate (0.02% w/v) were added and mixed. Finally, 2 mL of ascorbic acid (6% w/v) solution was added and mixed. The final concentration was maintained as 5 ppm for calculations. After 5 minutes of incubation at room temperature, the absorbance was measured at 650 nm with UV-Vis spectrophotometer (UV 2450, Shimadzu, Kyoto, Japan) and phosphorus concentration was determined from the standard curve (obtained from KH_2_PO_4_ as P - standard). The color was stable for more than one hour.

**Supplementary information- figures:**

**SI, Figure 1:**

**SI figure 1: Retention time of various organic acids**

**SI, Figure 2:**

**SI figure 2: Organic acid profiling for P-solubilization at different reaction time**

**SI, Figure 3:**

| **3a.**  **** | **3b.**  **** |
| --- | --- |

**SI, Figure 3: Response of cell growth with time due to change in P concentration during bioconversion of substrate to nano-hydroxyapatite. 3a. Change in biomass during P-solubilization with respect to the untreated control bacteria. 3b. Representative figure for equation 2**

**SI, figure 4:**

Well specifications:

Wells 1 to 8: Samples

1- 3 μg/mL

2- 6.25 μg/mL

3- 12.5 μg/mL

4- 25 μg/mL

5- 50 μg/mL

6- 100 μg/mL

7- 200 μg/mL

8- 500 μg/mL

9- solvent control (DI water)

10- Gentamycin (10μg/mL)

**
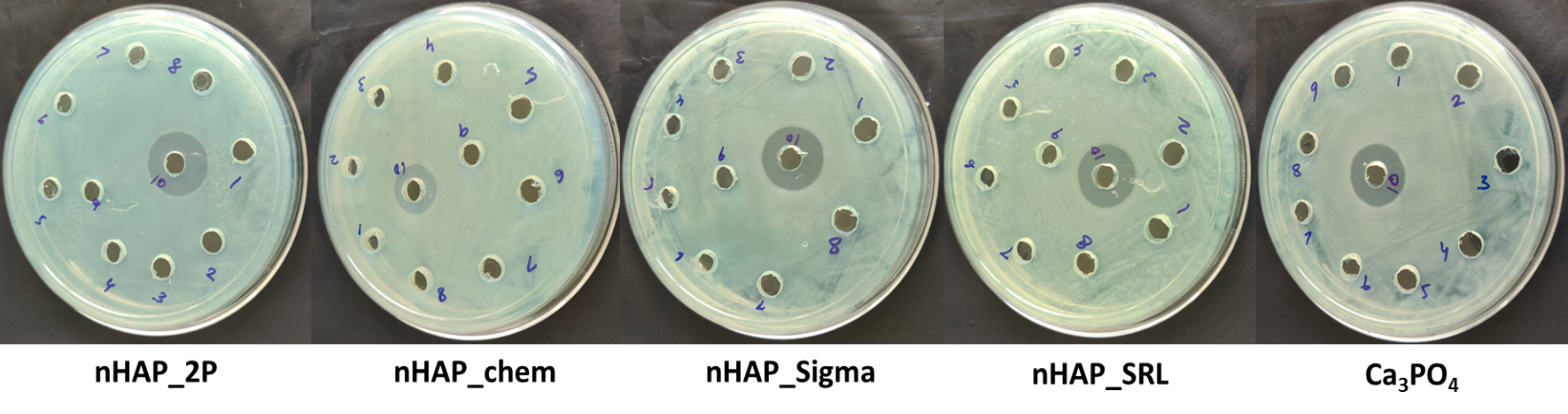
**

**
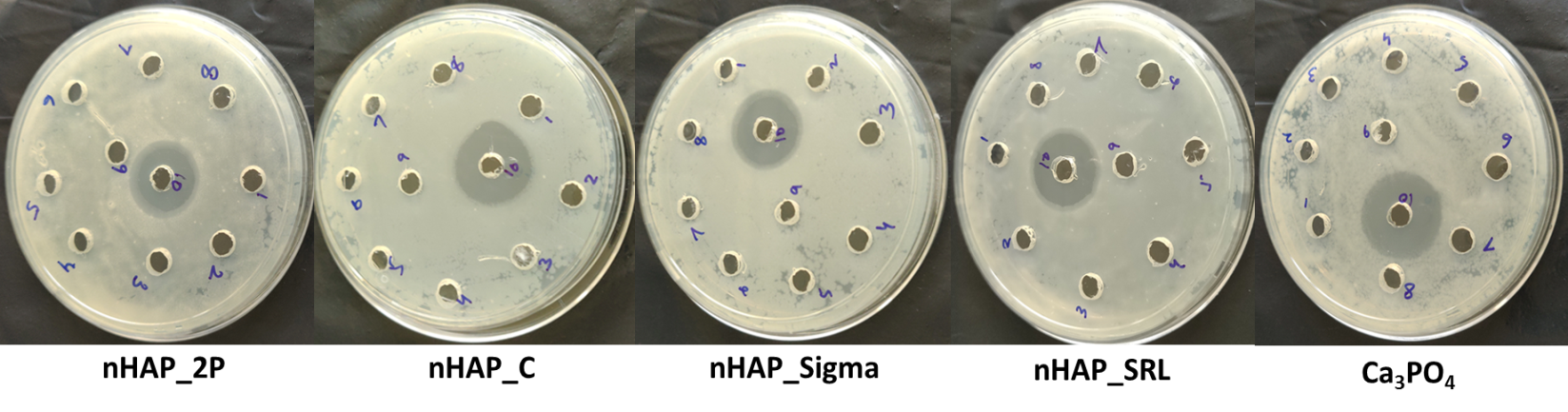
**

**
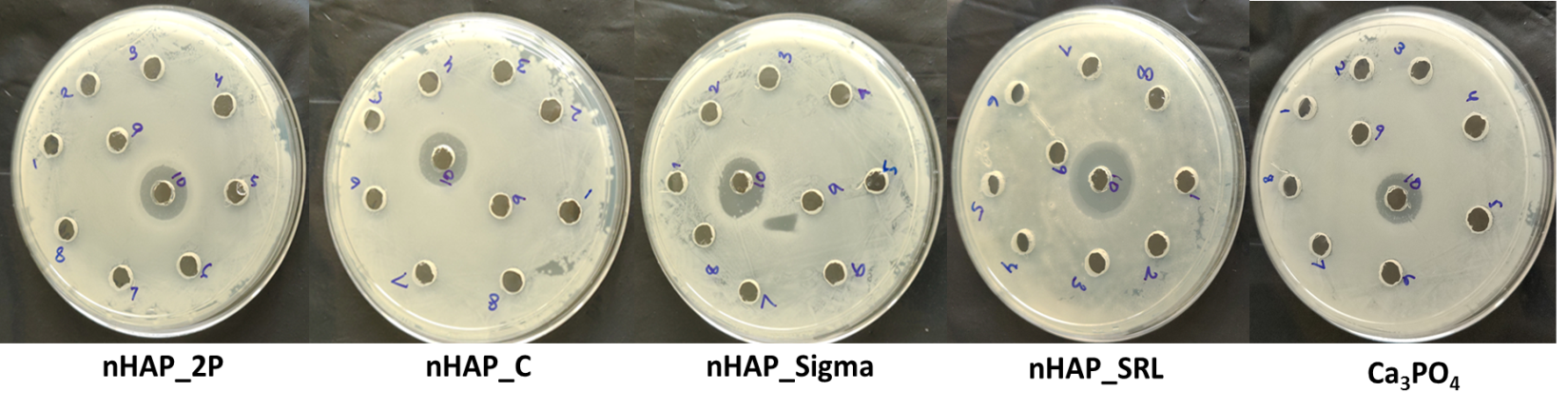
**

**
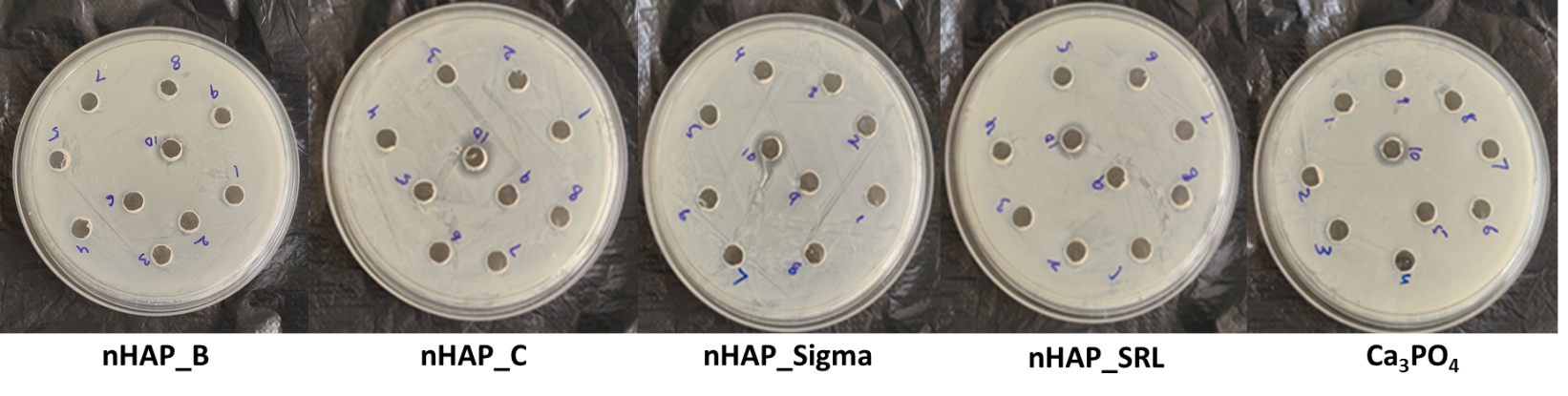
**

**
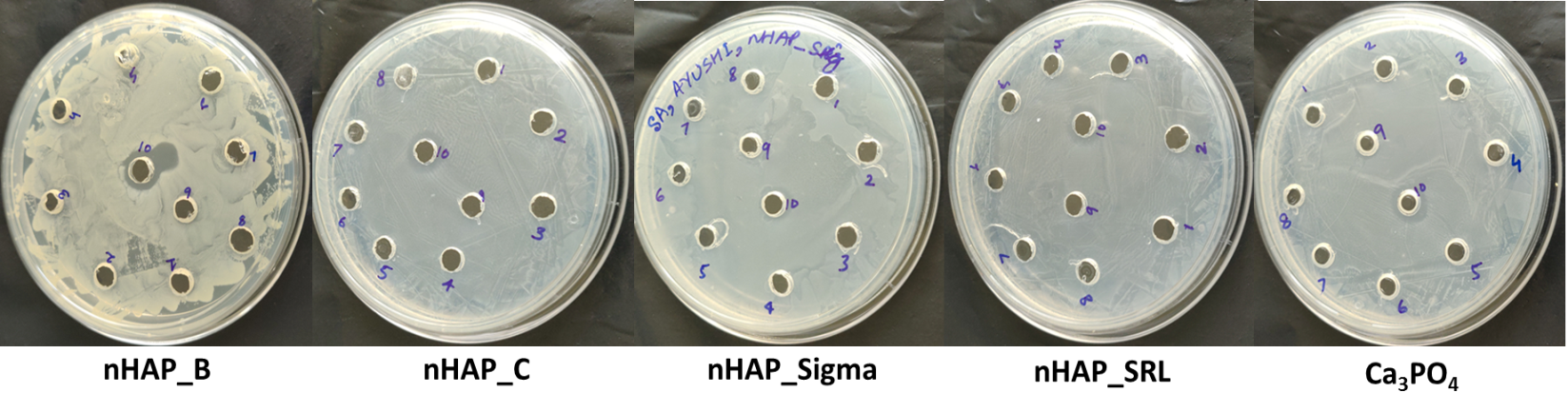
**

**SI figure 4:** **Zone of inhibition effect on a. *Pseudomonas aeruginosa*, b. *Bacillus subtilis*, c. *Escherichia coli*, d. *Acinetobacter baumannii* and e. *Staphylococcus aureus*; after treatment with different samples**

**Supplementary information- tables:**

**SI table 1: Linear regression equation from standard curve for various organic acids**

|  | Linear regression equation from standard curve | R^2^ |
| --- | --- | --- |
| Acetic acid | y = 1793.2x - 3837.2 | 1 |
| Citric acid | y = 113785x – 34174 | 0.9991 |
| Gluconic acid | y = 54452x + 10686 | 0.9949 |
| Maleic acid | y = 5E+06x – 171742 | 0.9996 |
| Oxalic acid | y = 896.61x - 3837.2 | 1 |
| Pyruvic acid | y = 36861x – 55887 | 0.9999 |
| Succinic acid | y = 3680.3x – 13754 | 0.9993 |
| Tartaric acid | y = 98919x + 41885 | 0.9977 |
